# Crystal Orientation Dynamics of Collective Zn dots before Preferential Nucleation

**DOI:** 10.1038/srep12533

**Published:** 2015-07-27

**Authors:** Chun-Chu Liu, Jun-Han Huang, Ching-Shun Ku, Shang-Jui Chiu, Jay Ghatak, Sanjaya Brahma, Chung-Wei Liu, Chuan-Pu Liu, Kuang-Yao Lo

**Affiliations:** 1 Department of Physics, National Cheng Kung University, Tainan 70101, Taiwan; 2 Department of Materials Science and Engineering, National Cheng Kung University, Tainan 701, Taiwan; 3 National Synchrotron Radiation Research Center, Hsinchu 300, Taiwan

## Abstract

The island nucleation in the context of heterogeneous thin film growth is often complicated by the growth kinetics involved in the subsequent thermodynamics. We show how the evolution of sputtered Zn island nucleation on Si(111) by magnetron sputtering in a large area can be completely understood as a model system by combining reflective second harmonic generation (RSHG), a 2D pole figure with synchrotron X-ray diffraction. Zn dots are then oxidized on the surfaces when exposed to the atmosphere as Zn/ZnO dots. Derived from the RSHG patterns of Zn dots at different growth times, the Zn dots grow following a unique transition from kinetic to thermodynamic control. Under kinetic-favoring growth, tiny Zn dots prefer arranging themselves with a tilted *c*-axis to the Si(111) substrate toward any of the sixfold in-plane Si<110> directions. Upon growth, the Zn dots subsequently evolve themselves to a metastable state with a smaller tilting angle toward selective <110> directions. As the Zn dots grow over a critical size, they become most thermodynamically stable with the *c*-axis vertical to the Si(111) substrate. For a system with large lattice mismatch, small volume dots take kinetic pathways with insignificant deviations in energy barriers.

Technologies involving heterostructure film growth are essential in governing the performance of many optoelectronic devices. Consequently, the mechanisms of heterostructure film growth have been extensively studied^1^,^2^,^3^. Nevertheless, these studies were overwhelmingly focused on epitaxial thin films with little being addressed on more complicated polycrystalline thin films. Heteroepitaxial thin films, involving the growth close to thermodynamic equilibrium, approach ideal conditions for modeling and are predominantly grown by molecular beam epitaxy or metal-organic chemical vapor deposition^4^,^5^,^6^. Under this scenario, tremendous efforts have been taken to understand the evolution of epitaxial film growth via various growth modes, such as Ge/Si and InAs/GaAs^7^,^8^,^9^. Conversely, polycrystalline thin film growth, lying far away from thermal equilibrium, is formidable to be studied due to uncountable kinetic factors involved. However, the visualization of growth kinetics is central to governing grain distribution and quality of polycrystalline thin films. Undoubtedly, the nucleation and coarsening of nuclei over a large area in the early stage must involve the competition between thermodynamics and kinetics locally, governing different morphologies, which still remain intractable^6^,^10^. Some experimental approaches only allow the examination of a local area without a systematical understanding of an entire sample, such as by transmission electron microscopy (TEM)^11^. Others only provide limited information on dislocations and misfit stress to analyze the relation of the island shape and size^12^,^13^,^14^,^15^. Comprehending a kinetic growth pathway, such as the evolution of the crystal orientation, for a polycrystalline film is systematically difficult^16^,^17^.

For a thin film starting with the island growth mode^18^,^19^, film growth mainly involves preferential nucleation, surface diffusion, and grain growth in sequence^17^. Preferential nucleation is one of the most frequently referred phenomena as an origin of the preferred orientation of a non-epitaxial film^20^,^21^,^22^. For example, in the growth of ZnO (Zn) on Si(111), where the lattice mismatch is 15.6% (30.6%) between *a*-axis of ZnO (Zn) and in-plane Si<110>, the preferential nucleation of ZnO (Zn) dots has been observed to assume ZnO (Zn) <0001>//Si<111>, although under a large lattice distortion. This distortion can be significantly relieved by defect formation of large-volume islands. Nevertheless, the large lattice mismatch plays a crucial role in the coherence of small volume islands with the Si(111) substrate^23^,^24^ and with one another. However, Zn or ZnO thin films deposited by magnetron sputtering as a widely-adopted growth in industry tend to be affected by numerous kinetic factors at relatively fast growth rates. It is our attempt to access such a complicated system by statistical ensemble methods to examine the evolution of sputtered film growth in early stage.

The primary nucleation of Zn dots or thin films grown by magnetron sputtering under kinetic conditions or conditions far away from thermal equilibrium is hardly investigated. This is mainly due to the limited availability of analytical methods that allow assessing the complex evolution of film growth in small volumes directly, especially during the nucleation period. By utilizing reflective second harmonic generation (RSHG), which is highly sensitive to surface dipolar structure^25^,^26^, we observe the anisotropic symmetrical distribution of dipoles of the Zn-O bonds on a ZnO surface^27^. For a *s*-polarized incident fundamental light, the *s*-polarized RSHG output light (called *ss*-RSHG) of ZnO{0002} planes exhibits a 3m symmetrical dipole (*a*_3_) for a ZnO surface with the *c*-axis normal to the substrate^27^. A 1mm symmetrical contribution (*a*_1_) comes into the RSHG pattern when the ZnO surface is tilted off-normal. For a dot system, the RSHG analysis gives a statistical result of crystalline orientations over all dots and presents a net dipole distribution. If the orientations of these dots are coherent, the RSHG pattern exhibits symmetric characteristics corresponding to the dipolar structure. On the other hand, the anisotropic contribution of the RSHG pattern is weakened for the random. Based on the advantage of RSHG in analyzing net dipole contribution of dots system, we develop a simple and non-destructive analytical method based on signals from reflective second harmonic generation (RSHG), allowing to probe the orientation distribution of surface islands over a large area.

In this work, we systematically study the growth mechanism of Zn/ZnO dots on Si(111) during nucleation by analyzing the RSHG patterns macroscopically. As an example, representing extreme kinetic growth conditions, the nucleation evolution of Zn dots on Si(111) by using magnetron sputter is attempted. The Zn/ZnO dot is constituted of a Zn dot grown with a thin ZnO shell of several atomic layers, which is formed upon exposure to the atmosphere through natural oxidation. The results are compared with 2D pole figure with synchrotron x-ray diffraction (XRD) to depict the statistical orientation distributions, and TEM analysis to provide at the atomic scale microscopically. The results are intriguing in that the *c*-axis of the Zn/ZnO dots start with possible off-normal orientations toward the sixfold in-plane Si<110> directions during nucleation, which then tilt to the normal direction of Si(111) at larger volumes. We show that a complete kinetic growth pathway of heterostructure film growth with a large lattice mismatch behaves far from thermodynamic predictions and can be studied by simply RSHG analysis. Due to the complexity of growth procedures in polycrystalline thin film growth, there is no universal theory at atomic scales to visualize the entire nucleation process yet. Through this work, we develop a new methodology, enabling to access to the complex and kinetically controlled growth processes closely. Besides, the existence of the complex metastable states between the dots and the substrate via kinetic pathways before complete relaxation to reach expected stable state will be a key reference for the growth of thin films by kinetic pathways.

## Results and discussion

### Size, density and composition of Zn/ZnO dots

Scanning electron microscopy (SEM) images (Supplementary, Fig. S1) reveal that the dots are uniformly distributed on the substrate. The size of the dots increases from ~11 nm at 10 min of deposition to ~81 nm at 60 min, whereas the density decreases from 1.4 × 10^11^ to 9.8 × 10^9^ with the increase in the growth time. The details of the size and density distribution with growth time are shown in Table S1 (supplementary information). Upon energetic particle bombardment on the substrate (the oxide layer was removed), the growth rate becomes slow approaching the thermodynamic regime, resulting in well-faceted dots or islands. The core of the dot is mainly composed of Zn and the ZnO shell is formed during later growth^28^.

### ss-RSHG theoretical analysis and experimental results of Zn/ZnO dots

The main nonlinear optical sources of the effective surface polarization from the Si(111) are the bulk electric quadrupoles and surface electrical dipole, both in a 3 m symmetry. When quantum dots exert stress on a Si(111) substrate, the 3 m symmetry signal is enhanced^29^,^30^.

**Figure 1:**
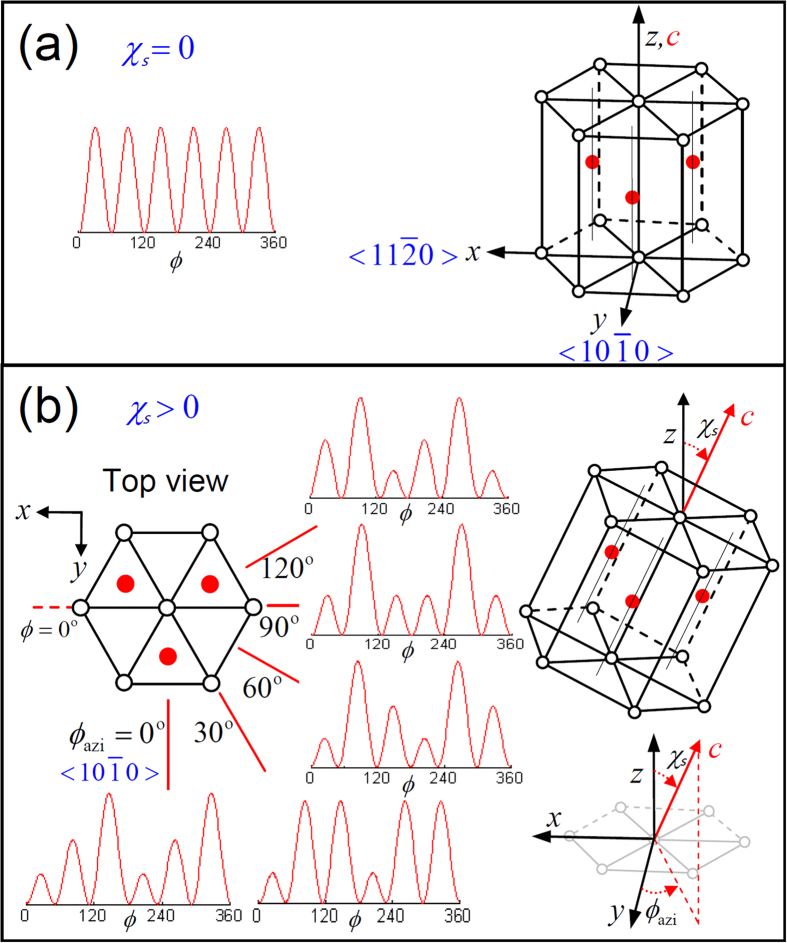
The correlation between the ss-RSHG pattern and the orientation of ZnO. crystal structure with *c*-axis (**a**) normal to substrate (*χ*_*s*_ = 0) and (**b**) away from the z-axis (tilting angle *χ*_*s*_ > 0) with with different azimuth angles, and their corresponding *ss*-RSHG intensity patterns.

The deposited quantum dots, either in the form of hcp Zn(0002) or wurtzite ZnO(0002), have a 6mm symmetry in bulk and a 3 m symmetry on the surface, in which the bulk 6 mm symmetry makes no contribution to the *ss*-RSHG intensity. The ss-RSHG intensity is mainly dominated from the ZnO shell of Zn/ZnO dots. Therefore, the *ss*-RSHG pattern of the material system comprising Zn/ZnO dots on Si(111) is 3 m symmetry with intensity *I*_*ss*_(2*ω*):


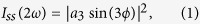


where *ϕ* is the rotational angle with *ϕ* = 0 relative to 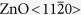 as shown in Fig. 1a, which exhibits the shape of six petals.

In Eq. (1), the *c*-axis of Zn/ZnO dots is assumed to be normal to Si(111), and the parameter *a*_3_ is proportional to the intensity of surface polarization from the Zn-O bonds^27^. Nevertheless, if the *c*-axis of Zn/ZnO dots tilts off-normal by an angle of *χ*_*s*_ at an azimuth angle of *ϕ*_azi_ (Fig. 1b), a rational transformation matrix (*M*^*t,a*^), expressed by


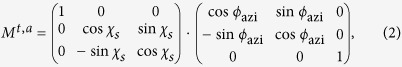


has to be added to the nonzero 3 m second-order susceptibility tensor (*χ*_*ijk*_). The second-order nonlinear polarization has the form: 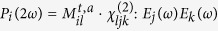, where *E*_*j*_ and *E*_*k*_ are the incident fundamental electric fields. By coordinate transformation, the correlated *ss*-RSHG intensity is given as


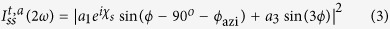


The first term on the right of the equation refers to 1mm anisotropic symmetry arising from the tilting of a ZnO crystal that breaks the crystalline symmetry. The azimuth angle *ϕ*_azi_ has correlations to the in-plane Si<110>, and the *ss*-RSHG pattern presents disparate size of petals for different *ϕ*_azi_ values. For example, Fig. 1b shows the patterns of *ϕ*_azi_ = 0°, 30°, 60°, 90°, and 120° with *χ*_*s *_= 20°. Therefore, the orientations of the crystalline dot arrays can be diagnosed from the anisotropic RSHG results by Eq. (3).

**Figure 2:**
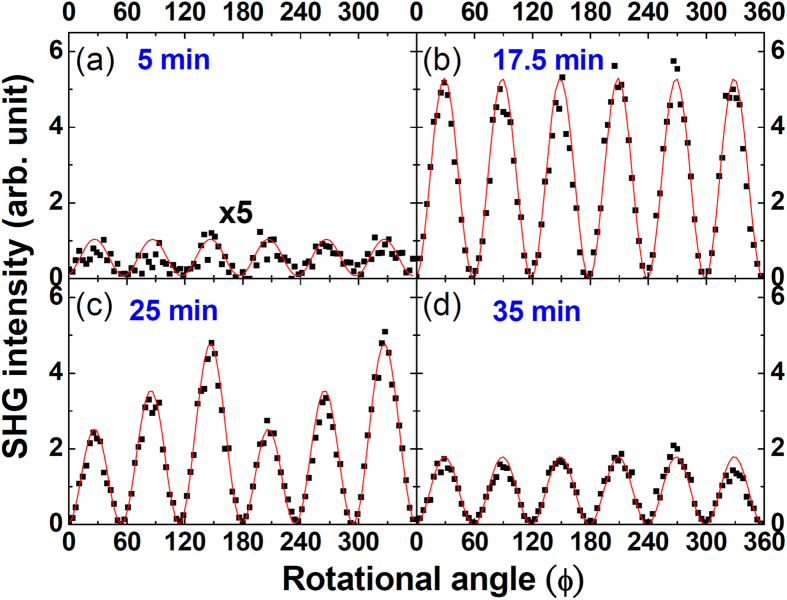
*ss*-RSHG intensity patterns of Zn/ZnO dots with different deposition times. The solid red line was plotted using the fitting parameters.

**Figure 3:**
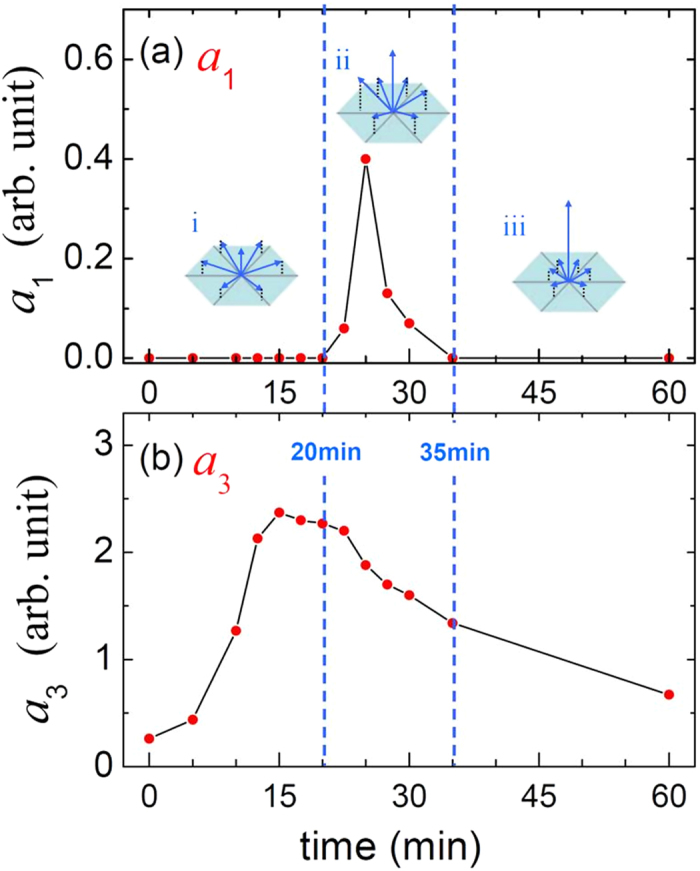
*ss*-RSHG parameters simulated from Eq. (3). (**a**) *a*_1_ and (**b**) *a*_3_ for different deposition times. The three insets in (**a**) depict the distribution of the *c*-axis orientation of Zn/ZnO dots.

The RSHG experimental setup is detailed in Ref. 31. The laser source was a pulsed Q-switched Nd-YAG laser with a wavelength of 1,064 nm, a pulse duration time of 6 ns, and a repetition rate of 10 Hz. The laser spot was not tightly focused to prevent damage to the sample. The detection area of each sample is about 10 mm^2^, which covers a large number of dots. The results of the optical *ss*-RSHG experiments reveal the anisotropic character and degree of coherence of these polar dots on a statistical basis. The *ss*-RSHG patterns of the Zn/ZnO dots grown on Si(111) are shown in Fig. 2 with the least squares curve fitting using Eq. (3) in red solid lines. The similar analyses for Zn/ZnO dots grown on Si(100) has no any symmetrical RSHG pattern and their RSHG signals are tiny (<0.1 same scale as Fig. 2). Since the surface symmetry of Si(100) is 4 mm that is not the same type as the surface symmetry of Zn, there is no any strain between Zn and Si(100), and hence no coherence between the structure of Zn and Si(100). The results reflect to the coherent growth between Zn and the substrate should be based on the same type of crystalline symmetry as least. The fitted parameters *a*_1_ and *a*_3_ are plotted as a function of growth time in Fig. 3. The value of *a*_1_ starts with zero until the growth time of 20 min, and then peaks at 25 min. Subsequently, *a*_1_ gradually returns to zero at 35 min growth time and remains zero thereafter. The results reveal an unusual growth phenomenon in which epitaxial dots tilt their crystallographic orientations during growth, implying a large lattice mismatch. To interpret the evolution of *a*_1_, the following scenarios are speculated (the associated possible crystal orientations are drawn in Fig. 3). (*i*) When the growth time is less than 20 min, the *c*-axes of the dots are tilted off-normal with the azimuth directions evenly distributed among in-plane Si<110>, rendering the *a*_1_ values cancelled out by each other. Besides, this sixfold tilting of the dots still makes a constructive contribution to *a*_3_, as shown in Fig. 3b. The *a*_3_ value reaches the maximum at 15 min of growth and starts to decrease thereafter, corresponding to when the dots increase to the critical volume and then start to lose their coherency for the happening of stress relaxation. (*ii*) When the growth time is between 20 and 35 min, *a*_1_ is non-zero but *a*_3_ keeps decreasing. When the dot volume increases, the lattice coherency keeps losing as the decreasing trend of *a*_3_. Correspondingly, the dots reorient themselves toward a more symmetric geometry to minimize the surface energy as normal to Si(111). During this process, the accumulative *c*-axes of the dots have asymmetrically projected orientations on some of the sixfold in-plane Si<110>, causing a nonzero resultant *a*_1_. The parameter *a*_1_ soon reaches the maximum of 0.4 for 25 min-grown Zn/ZnO dots, representing the stable kinetic configuration with *χ*_*s*_ ≠ 0 and *ϕ*_azi_ = 0° deduced from the *ss*-RSHG pattern showing periodic step-up petals (Fig. 2c). This *a*_1_ is not contributed from grain boundaries^27^ since *ϕ*_azi_ should be 30° in the grain boundary case. (*iii*) When the growth time is longer than 35 min, *a*_1_ = 0 and *a*_3_ keeps decreasing. This phenomenon implies that most dots have the *c*-axis perpendicular to Si(111) for the thermodynamic stable configuration. The RSHG parameters of *a*_*1*_, *a*_*3*_ and *χ*_*s*_ reflect the net dipole contribution from Zn/ZnO dots covered by irradiated area, which reveals the coherent growth and the evolution of crystal orientation of the collective Zn dot system during growth. These results would be a guide to do further analyses.

The Zn/ZnO dots contribute dominantly to the RSHG intensity due to the surface polarization of Zn-O. For example, the anisotropic coefficient *a*_3_ in Fig. 3b of the 17.5 min Zn/ZnO dots is higher than pure Si(111) by more than 10 times. According to the bond orbital theory, the existing bonding distortion in the interface of Zn dots and Si(111) cannot cause this large enhancement as well as the SiO_x_ layer^29^,^30^,^31^,^32^. The optical RSHG signal sums over the entire area of all Zn/ZnO dots being probed.

On the other hand, the tilting 6 mm structure of the Zn(0002) bulk may also contribute the 3m and 1mm components to *I*_*ss*_, which has the form 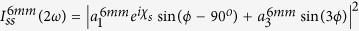, similar to Eq. (3) but independent on the azimuth angle *ϕ*_azi_. Tilting Zn(0002) bulk has an un-eliminated 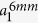 component from dots because this 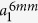 component constructively collects from all azimuth angles. This result is in conflict with that in region (*i*). The 6 mm *ss*-RSHG intensity from bulk Zn(0002) in tilting condition is too small to be considered here.

### 2D pole figure experiment and analysis of Zn/ZnO dots

**Figure 4:**
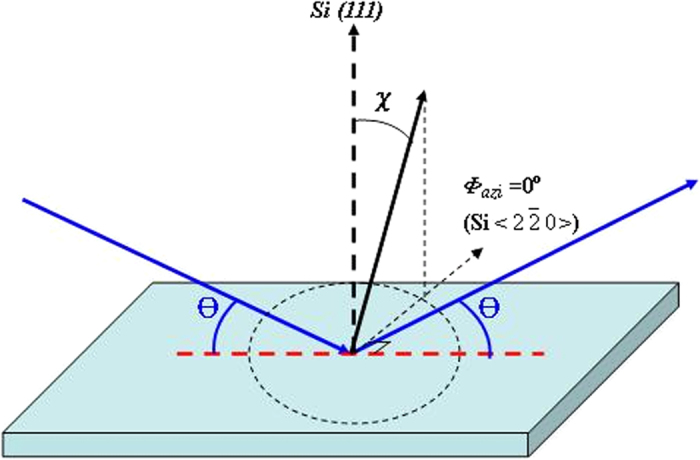
Diagram of 2-D pole figure measurement.

The above RSHG speculation can be confirmed by 2D pole figure and TEM analyses. Pole figure that plotted in polar coordinates consisting of the tilt and rotation angles is a power tool to estimate the orientation distribution of crystal^33^. A pole figure is measured at a fixed scattering angle and consists of a series of Φ scans (in- plane rotation around the center of the sample) at different tilt (*χ*) or azimuth 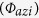 angles, as shown in Fig. 4 for clarifying the graphical representation of the orientation of objects in space. As compared with 2-D Bragg X-ray diffraction pattern, which shows diffraction signals in a plane along theta and 2-theta axis, the pole figure could provide detailed information for orientation distribution of sample in real space^34^. However, pole figure measurement is hard to be performed in the dots system. In this work, the pattern of 2D pole figure could be obtained through long-time measurement with synchrotron x-ray source. The photon energy of the Synchrotron XRD was 8 keV (wavelength = 1.54982 Å) with a flux estimated to be 10^11^ photons/s. To estimate the overall orientation distribution of Zn islands, pole figures of 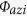 (ranging from 0° to 360°) and *χ* (ranging from 0° to 88°) scans with the 2*θ* set at Zn(0002) are made by synchrotron X-ray source. The azimuth 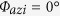 aligns with the orientation of 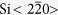.

**Figure 5:**
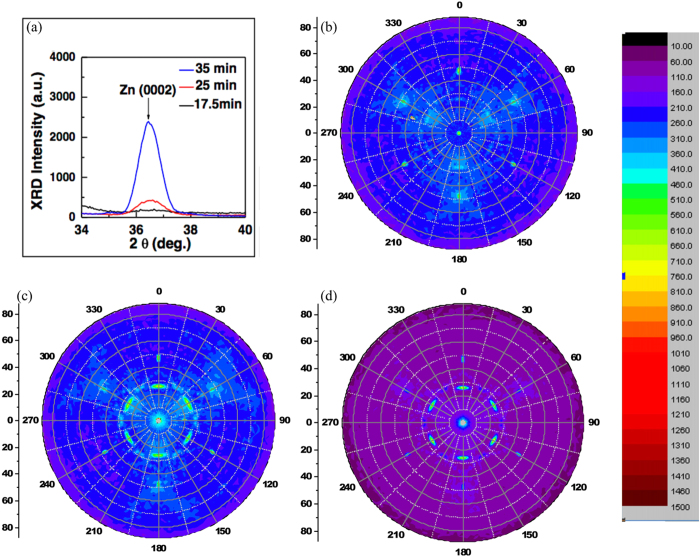
Synchrotron XRD and 2D pole figure analysis of Zn/ZnO dots. (**a**) Synchrotron XRD results of Zn dots with the deposition time of 17 min, 25 min and 35 min, (**b**) 2D pole figure of Zn dots with the deposition time of 17.5 min, (**c**) 25 min and (**d**) 35 min.

Figure 5a shows the XRD pattern (*θ*–2*θ* scan scan) of Zn/ZnO dots. The intensity of the Zn(0002) peak increases with the growth time and the peak position shifts slightly towards lower Bragg angles as 36.65°, 36.60°, and 36.45° for 17.5, 25, and 35 min, respectively, as a consequence of (tensile) strain relaxation (bulk Zn: 2*θ*_Zn(0002)_ = 36.29°). The patterns of 2D pole figure at *θ* of 36.65°, 36.60°, and 36.45° (the peak of Zn(0002)) are shown in Fig. 5(b–d). The pole figure of Zn(0002) diffraction angle with growth time 17.5 min (Fig. 5b) exhibit symmetry orientation distribution. This indicates that Zn dots grow on Si(111) and behave symmetry orientation distribution of Zn crystal. The pattern at *χ* = 48° and *χ* = 28° behave two-groups of threefold symmetry with broad features as well as another narrow feature of *χ* = 48° with 60° rotation respect to two-groups, which reveals that Zn dots are strained by Si(111) substrate with less relaxation since the intensity of the Zn(0002) peak is not strong. Besides, the peaks at *χ* = 48°, and 28° are found to correspond to the dots with 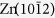, and 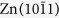, respectively, parallel to the substrate Si(111). With longer growth time of 25 min, the intensity of the Zn(0002) peak is increasing, as shown in Fig. 5c. The intensity at *χ* = 0°, and 28° is getting stronger respect to a 17.5 min sample. It means partly Zn dots were relaxed from the strain state and turn it’s orientation parallel to the direction of Si(111). When the growth time increases to 35 min, the pattern at *χ* = 0° displayed in Fig. 5d become much stronger, which illustrates that Zn dots with the longest time of growth exhibit strong concentrated behavior of orientation Zn(0002) to *χ* = 0°. Besides, the rocking curve (shown in Figure S2 in the supplementary materials) of the pattern at *χ* = 0° has a narrow full width at half maximum (FWHM) of 0.305°. Rocking curve analysis usually provides valuable information about the size of coherently diffracting domain (particle/grain size) and the distribution of different orientation from each dot. In this case, such a low value of 0.305 mainly comes from the misorientation of the dots, which indicates that the dots have high preferred orientation.

### The correlation between 2D pole figure and ss-RSHG

The analyses of the 2D pole figure support the results of ss-RSHG pattern. The *ss*-RSHG result of Zn/ZnO dots (Fig. 3) indicate that the Zn/ZnO dots with growth time of 17.5 min exhibit Zn dots grown coherently on Si(111) with a tilted c-axis to Si(111) substrate toward any of the threefold in-plane Si<110> equally. Thus, the *a*_1_ value is vanished and the 3 m symmetrical dipole contribution is attributed constructively to *a*_3_, which corresponding to the dots with lower population in the direction of Zn(0002), but being coherently strained by Si(111). The value of a_3_ for Zn/ZnO dots of 25 min and 35 min is getting smaller since the intensity of Zn(0002) in the pole figure is getting strong. It means that larger Zn dots would relax from the strain of in-plane Si<110>and are not coherent with in-plane Si<110>. Besides, the value of a_1_ is not zero in the case of 25 min due to the asymmetry intensity distribution at some of the in-plane Si<110> and can’t be cancelled. Those results from 2D pole figure provide strong evidence for the illustration of the *ss*-RSHG pattern. RSHG result reflects to the net dipole contribution of Zn/ZnO dot system and is complementary to XRD analysis, which is generated from the sum total of Zn dots including different size of dots. The inset of Fig. 3a illustrates the evolution of the crystal orientation of Zn/ZnO dots grown on Si(111) with the growth time (size). Therefore, 2D pole figure shows the result of all Zn dots with varying size. However, the 2D pole figure performed with synchrotron X-ray is huge expensive and wastes time. Based on the above analyses, RSHG method to study the evolution of Zn/ZnO dots is convinced to be a relied and simple way.

### TEM analysis and diffraction patterns (DP) of Zn/ZnO dots

**Figure 6:**
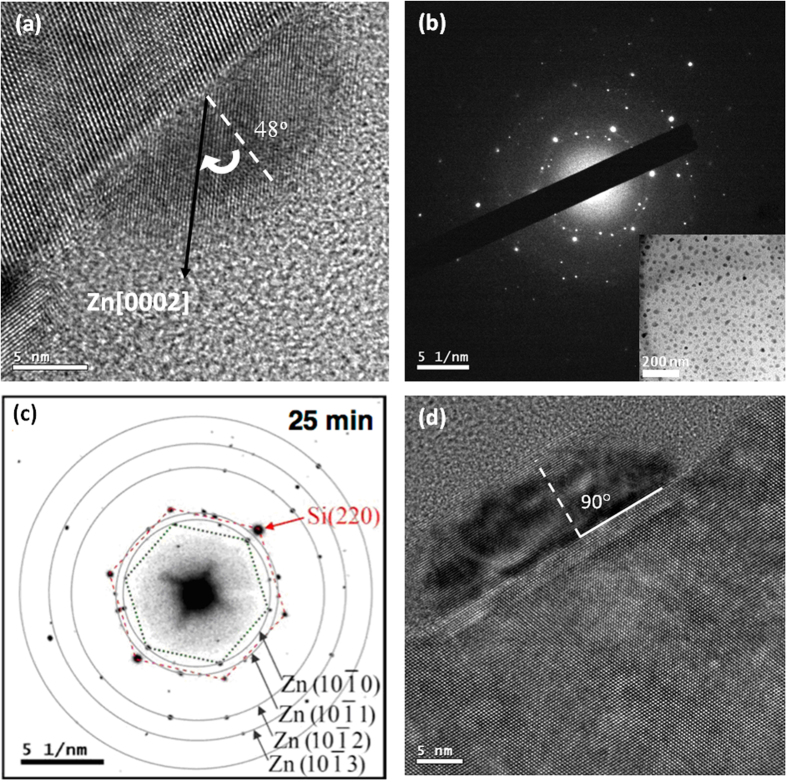
HRTEM and DP of Zn/ZnO dots. (**a**) Cross-sectional HRTEM for 17.5 min. (**b**) DP for the deposition times of 17.5 min. The insert shows the plan-view TEM image. (**c**) DP for the deposition times of 25 min (the intensity has been inverted from black to white). (**d**) Cross-sectional HRTEM for 35 min.

The cross-sectional TEM images were taken along Si<110> zone axis, while the plan-view images and DP were taken along Si<111> zone axis. The high resolution TEM (HRTEM) images demonstrate good single crystalline structure for the Zn/ZnO dots. The dots grown for 17.5 min exhibit random orientations, where four different orientations were observed from 17 dots. For example, the *c*-axes of the Zn dot in Fig. 6a is found to be *χ* ≈ 48°. To crosscheck the *a*-axis orientation of the dots, we performed DP in Fig. 6b from a plan-view sample (image in the inset). This pattern only exhibits selected correlations between the dots and substrate. A better dot/substrate correlation is found for the 25 min sample. The DP from a plan-view image as shown in Fig. 6c reveals that the Si{220} planes (red dashed line) are parallel to 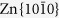 planes (green dot line). There are two rings on the diffraction pattern can be assigned to 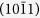 and 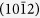 for hexagonal Zn crystal. The result agrees with analyses of the pole figure in Fig. 5c. In the case of the 35 min-deposited sample, mostly dots exhibit *c*-axial direction normal to the substrate through HRTEM observation, as shown in Fig. 6d. Unlike the epitaxial growth due to a small lattice mismatch, this growth follows pattern matching growth by establishing only the orientation relationship between substrate and dots analyzed and described above. Therefore, the incoherent interfaces are certainly composed of point defects and dangling bonds to accommodate the large mismatch, causing the dots almost completely strain relaxed. However, the point defects are difficult to be analyzed by TEM but no other defects are present at and across the interfaces. Besides, no strong strain contrast around dots in either bright-field or dark-field TEM images can prove the incoherent interface.

### The orientation evolution of Zn/ZnO dots

The crystal orientation is determined by the competition between strain energy minimization and surface energy minimization^16^. The strain energy is proportional to the square of mismatch strain^19^. The fact that the dots evolve with different orientations and even more than one orientation co-exist at one growth condition strongly suggest it is a kinetic process through many intermediate metastable states mainly determined by the strain via chemical potential. In the early stage, under the diffusion-limited regime for insufficient thermal energy provided, the chemical potential driving diffusion of adatoms is presumably determined by local size mismatch. Then, small volume dots take many kinetic pathways with insignificant deviations in energy barriers. This leads to the tilting of the *c*-axis of Zn dots on the substrate surface with sixfold anisotropic distribution, and further tend to tilt small angle to one of in-plane Si<110> as reaching the metastable state. With the increased size of the Zn dots, the surface free energy decides the crystalline orientation when the lattice strain has been relaxed. The orientation Zn[0001] is chosen because the surface free energy of (0001) is larger than those of 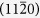 and 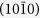
35,36. The dots with the hemispherical shape in this orientation has almost no (0001) plane exposed, and represents the most symmetric shape for reducing surface energy.

Zn dots are solidified from different liquid volume given by the growth time, and the dot distribution is probably determined by surface diffusion of massive atoms. Thereby, unlike in the epitaxial system, the orientation and distribution of entire dot ensemble is determined by the kinetic pathway since the speed of solidification is so fast, which reveals the existence of metastable states as a result of solidification.

## Conclusion

As the composition of Zn/ZnO dot is a Zn dot covered by ZnO shell, the analyses of the evolution of Zn/ZnO dots can be performed by nonlinear optics and material analyses (XRD and TEM) simultaneously. We have successfully developed a methodology to study the evolution of surface dots by using RSHG patterns with which sputter growth of Zn/ZnO dots under specific conditions by introducing hydrogen and substrate bias is explored as an example. Both synchrotron XRD and TEM results are in full support of the observation of the orientation evolution before preferential nucleation of Zn dots. In the early stage, the c-axis of Zn dots are tilted off the substrate normal, but with sixfold anisotropic distribution and then gradually tilted toward the substrate normal with smaller tilting angles to in-plane Si<110> directions. Eventually, when the dot volume exceeds a critical size, the c axes of all the dots are oriented to be parallel to the substrate normal determined by surface energy. The RSHG methodology can be extended to study dot formation in real time and used as a complimentary tool for more time-consuming analysis of pole figure by synchrotron XRD. Consequently, the main contributions of this work are to systematically analyze the kinetic pathway of the dot growth and to reveal the existence of meta stable states before preferential nucleation.

## Methods

### Sample preparations

The Zn dots were grown on 4 in. Si(111) substrates by radio frequency (RF) magnetron sputtering with an RF power of 150 W and a mixed gas comprising H_2_ and Ar (H_2_ < 20%) under a substrate bias of −500 V at an elevated temperature of 445 °C. Si(111) substrate was treated with a standard RCA clean before sputtering. The ZnO target was of high purity (99.99%). The base and working pressures were 3 × 10^−6^ and 1 × 10^−2^ Torr, respectively^28^. The series of dots for characterization were grown for 5–60 min. The Si(111) surface was further purified during sputtering by bombardment of accelerated Ar^+^ ions and H_2_ reduction. The substrate bias applied on the Si substrate leading to a huge amount of energetic ionized Ar^+^ and ZnO^+^, accelerated by the negative substrate bias to bombard the silicon substrate. Therefore, the energetic ions with higher energy can diffuse across surface easily to favorable sites, while etching the native SiO_2_ layer away. However, if supplied too much as in this case, the energetic Ar^+^ ions can easily break ZnO bonding with the bonding energy of ~3 eV, leaving behind mostly metal Zn-rich phase, which is liquid-like at the growth temperature^28^. Hydrogen flux was introduced to eliminate most of the residual oxygen gases to clean the surface as well as stop defective nuclei from growing in a reductive atmosphere^37^. Therefore, the crystallinity of Zn dots can be improved owing to the enhancement of surface diffusion of atoms under the synergy effect of hydrogen treatment and substrate bias^28^. Zn dots solidify from the liquid phase after cooling and removing from the chamber. The evolution of preferential nucleation of Zn dots can be influenced by all growth variables, such as sputtering power, the substrate bias and H_2_ partial pressure. Even more complicated, these variables can modify Si(111) substrate surface, which also serves as another kinetic factor for the subsequent growth. All these factors cause the complete analysis of film growth a daunting task and only one set of condition is selected in this work as stated.

Besides, the growth proceeds under oxygen-deficient conditions owing to the physical bombardment and H_2_ reduction. The cores of the dots are mainly composed of Zn and the ZnO shell is formed during later growth through the natural oxidation. The evolution of Zn/ZnO dots can be analyzed specifically by nonlinear optics (mainly contributed from ZnO shells) and by other materials characterizations tools such as XRD and TEM (for Zn cores) simultaneously.

## Additional Information

**How to cite this article**: Liu, C.-C. *et al*. Crystal Orientation Dynamics of Collective Zn dots before Preferential Nucleation. *Sci. Rep*. **5**, 12533; doi: 10.1038/srep12533 (2015).

## Supplementary Material

Supplementary Information
