# Supplementary material for: Crystal Orientation Dynamics of Collective Zn dots before Preferential Nucleation
Source: Sci Rep. 2015 Jul 27;5:12533. doi: 10.1038/srep12533 (PMC4515603; doi:10.1038/srep12533)
Supplement: Supplementary Information [file srep12533-s1.pdf]

**Supplementary Information**

# Crystal Orientation Dynamics of Collective Zn dots before Preferential Nucleation

Chun-Chu Liu[1], Jun-Han Huang[2], Ching-Shun Ku[3], Shang-Jui Chiu[3], Jay Ghatak[2], Sanjaya Brahma[1], Chung-Wei Liu[1], Chuan-Pu Liu[2], & Kuang-Yao Lo[1]

[1]Department of Physics, National Cheng Kung University, Tainan 70101, Taiwan

[2]Department of Materials Science and Engineering, National Cheng Kung University, Tainan 701, Taiwan

[3]National Synchrotron Radiation Research Center, Hsinchu 300, Taiwan

**Table S1:** Zn/ZnO dot size and density distribution with growth time

| | Density ($/cm^2$) | Average dot size (nm) |
|---|---|---|
| 10 min | $1.4\times10^{11}$ | 11.4 |
| 12.5 min | $9.4\times10^{10}$ | 17.2 |
| 17.5min | $7.8\times10^{10}$ | 21.7 |
| 25 min | $4.7\times10^{10}$ | 35.3 |
| 27.5 min | $3.2\times10^{10}$ | 37.7 |
| 35 min | $2.3\times10^{10}$ | 44.3 |
| 60 min | $9.8\times10^{9}$ | 80.8 |

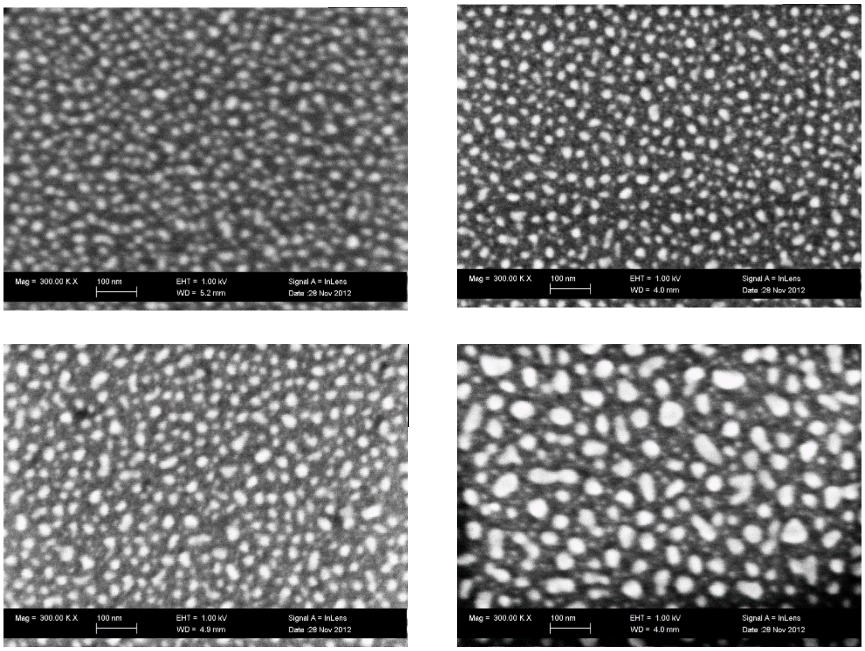


**Figure S1**. FE-SEM image of Zn/ZnO dots for different growth time (a) 12.5 min (b) 15 min (c) 17.5 min (d) 27.5 min

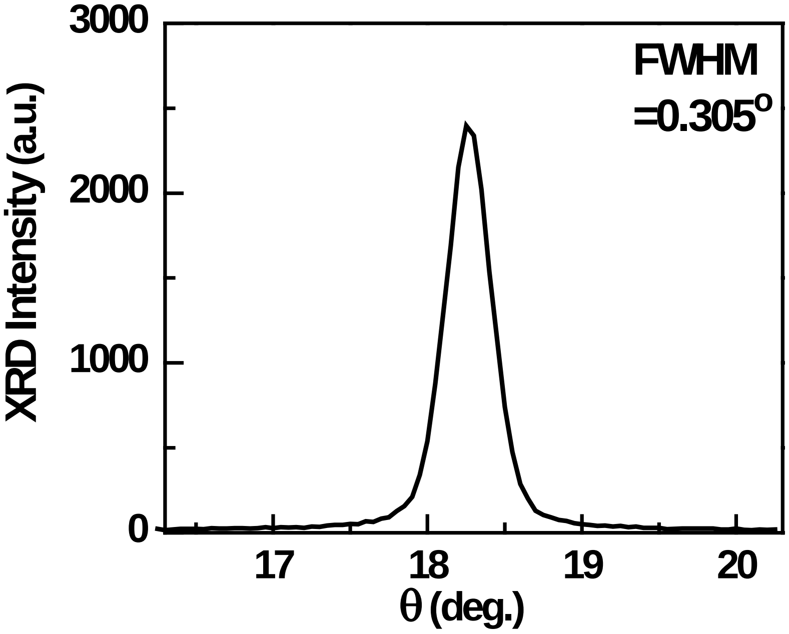


**Figure S2.** Rocking curve of Zn dot grown on Si(111) at the growth time of 35 min.
